# Integrated Transcriptomic and Metabolomic Analysis Elucidates the Impact of Acute Ammonia Stress on Carbohydrate and Lipid Metabolic Pathways in Yellow Catfish (*Pelteobagrus fulvidraco*)

**DOI:** 10.1155/anu/5545977

**Published:** 2025-11-10

**Authors:** Xue Li, Shidong Wang, Muzi Zhang, Ming Li, Chao Chen

**Affiliations:** ^1^Key Laboratory of Animal Genetics, Breeding and Reproduction in the Plateau Mountainous Region, Ministry of Education, Guizhou University, Guiyang, Guizhou 550025, China; ^2^College of Animal Science, Guizhou University, Guiyang 550025, China; ^3^College of Biosystems Engineering and Food Science, Zhejiang University, Hangzhou 310000, China; ^4^School of Marine Sciences, Ningbo University, Ningbo 315211, China

**Keywords:** ammonia stress, glycogenolysis, lipolysis, ureagenesis, yellow catfish

## Abstract

Ammonia stress (AS) constitutes a significant environmental challenge that impedes aquaculture development. In this investigation, histomorphology assessments, physiological, and biochemical parameter analyses, and multiomics approaches were employed to elucidate the impact of acute AS on yellow catfish (*Pelteobagrus fulvidraco*). Findings indicated that serum ammonia concentrations exhibited a dose-dependent increase, correlating with the intensity and duration of stress. As the primary detoxification organ, the liver facilitates ammonia clearance by upregulating genes involved in glutamine and ureagenesis (glutamine synthase [*gs*], carbamoyl-phosphate synthase [*cps*], ornithine transcarbamylase [*otc*], argininosuccinate lyase [*asl*], argininosuccinate synthase [*ass*], arginase [*arg*]), thereby promoting glutamine and ureagenesis while consuming glutamate, argininosuccinic acid, aspartic acid, arginine, and adenosine triphosphate (ATP). Physiological and biochemical data revealed that AS significantly elevated serum glucose, liver triglyceride (TG), and total cholesterol (TC) levels. Histological examination demonstrated a marked reduction in liver glycogen stores alongside a progressive accumulation of lipid droplets proportional to stress severity, suggesting activation of liver glycogenolysis coupled with suppression of lipolysis. Integrative transcriptomic and metabolomic analyses indicated a reprograming of liver energy metabolism characterized by enhanced glycogenolysis and suppressed lipogenesis: liver glycogen content decreased, key glycolytic gene expression (*hk1*, *pdhx*) was downregulated, and tricarboxylic acid (TCA) cycle flux was diminished due to decreased *cs* expression. Concurrently, transcription of fatty acid β-oxidation enzymes (*acsbg1*, *cpt1*) was suppressed, leading to palmitic acid accumulation and impaired lipid-derived energy production. Nonetheless, reorganization of carbon flux through upregulation of *mdh2* and *idh1* facilitated pyruvate utilization in the TCA cycle, promoting NADH generation and sustaining oxidative phosphorylation, as evidenced by increased ATP turnover and content. This study elucidates the metabolic response to AS via increased glycogenolysis. Optimizing liver glycogen reserves serves as a nutritional strategy to enhance ammonia tolerance. Targeted regulation of key genes (*pygl*, *pk*, *mdh2*, *idh1*) to promote glycogen–pyruvate metabolism may mitigate ammonia toxicity effects and improving aquaculture productivity.

## 1. Introduction

Ammonia in aquatic systems originates from both natural and anthropogenic sources. Natural sources encompass the excretion of nitrogenous waste by organisms and microbial nitrate reduction in anaerobic conditions. Anthropogenic inputs include agro-industrial activities and sewage effluent. The anticipated proliferation of ammonia as a low-carbon energy vector could precipitate a hundredfold increase in global production, thereby amplifying the risk to aquatic fauna [1]. Elevated ammonia (NH_3_) concentrations compromise cellular integrity by disrupting mitochondrial proton gradients and inhibiting adenosine triphosphate (ATP) synthesis, consequently impairing cellular energy metabolism [2, 3]. Furthermore, ammonia exposure can trigger the accumulation of reactive oxygen species (ROS), resulting in liver steatosis, gill epithelial hyperplasia, and erythrocyte damage [4, 5], and induce gill structural and functional damage [6], ultimately leading to behavioral anomalies such as lethargy, surface aggregation ("floating head”), and cessation of feeding [7, 8]. Acute ammonia exposure can elevate fish mortality rates, while chronic exposure can retard growth and weight gain. For instance, cyprinid fish exposed to sustained high ammonia concentrations may experience growth rate reductions exceeding 50% [5, 9, 10].

Given that ammonia can impede fish growth performance, it is inevitable that ammonia will impact the nutrient metabolism profile of fish. Carbohydrates are the key energy nutrients in the fish diet and occupy the core position of preferential decomposition in the body's energy supply [11]. As the lowest cost energy source, reasonable intake of carbohydrates can not only significantly promote fish growth but also effectively reduce the cost of aquaculture by optimizing metabolism and reducing ammonia excretion [12–14]. However, fish have significant limitations in their ability to utilize carbohydrates, and long-term excessive intake of carbohydrates can cause growth arrest, fat accumulation, and nutrient metabolism disorders [15]. Previous studies have shown that ammonia stress (AS) can activate the glycolytic pathway, promote glycogenolysis, and enhance lactate production and the expression of related genes in hybrid grouper (*Epinephelus fuscoguttatus*♀ × *E. lanceolatus*♂) [16]. The Chinese striped-neck turtle (*Mauremys sinensis*) and juvenile oriental river prawn (*Macrobrachium nipponense*) inhibited gluconeogenesis to save energy while promoting glycolysis under AS by activating AMPK signaling pathway, thus preferentially meeting energy demands under stress conditions [17]. The regulation patterns of glucose metabolism in different species show significant interspecific heterogeneity under AS, which may be closely related to the divergence of evolutionary adaptation strategies and physiological metabolic characteristics of species.

Lipid metabolism is crucial for fish physiological functions, encompassing energy homeostasis, cellular structure integrity, growth, development, immune responses, and reproduction [18]. Some studies have found that ammonia nitrogen stress can interfere with fatty acid synthesis and oxidation, activate the arachidonic acid metabolic pathway, and induce lipid peroxidation in gill tissues of juvenile four-finger threadfin (*Eleutheronema tetradactylum*). These changes are not only the result of damage caused by stress but also the active response of the body to adapt to adversity by adjusting lipid composition and metabolic pathways. However, long-term or high-intensity AS can lead to irreversible lipid metabolism disorder, affecting its growth and survival [19]. Furthermore, elevated ammonia concentrations (>30 mg/L) significantly suppressed the expression of lipid synthesis-related genes and stimulated lipolysis in the muscle of *Oncorhynchus mykiss*, leading to substantial depletion of PUFAs (particularly *n−*3 PUFAs), increased lipid oxidation, and compromised muscle quality [20]. In Largemouth Bass (*Micropterus salmoides*), AS primarily inhibited both lipid synthesis and degradation, an adaptive strategy that may exacerbate liver damage and metabolic disorders [21]. Moreover, AS directly inhibited linoleic acid and arachidonic acid metabolism in the hepatopancreas of *Litopenaeus vannamei*, triggering a cascade of insufficient energy production, lipid deposition, and oxidative damage, ultimately reducing ammonia tolerance and increasing mortality in shrimp [22]. In conclusion, the regulatory effects of ammonia on lipid metabolism in aquatic organisms exhibit significant heterogeneity, influenced by ammonia exposure concentration, stress duration, and species-specific factors.

As a significant freshwater economic species, yellow catfish holds a crucial position in aquaculture. While research on the glucolipid metabolism of other fish species, such as hybrid grouper, rainbow trout, and largemouth bass, under AS has advanced, metabolic regulation patterns vary due to differences in evolutionary adaptation strategies and physiological metabolic characteristics among different species. The exceedance of ammonia in the water increases the susceptibility of pathogenic bacteria and causes serious economic losses in yellow catfish cultivation. The glucolipid metabolic patterns of yellow catfish under AS remain unclear. Investigating its glucolipid metabolic regulation under AS is essential for understanding the physiological mechanisms of yellow catfish in response to AS, elucidating the internal causes of growth inhibition, optimizing nutritional regulation strategies in aquaculture to reduce ammonia excretion and production costs, and ensuring the healthy growth and improved aquaculture efficiency of yellow catfish.

## 2. Materials and Methods

### 2.1. Experimental Fish and Acclimatization

The experimental fish were procured from an aquaculture facility in Jiaxing, Zhejiang, and temporarily held within a water recirculation system at the Ningbo University aquaculture base. The fish were fed commercial feed twice daily (7:00 and 18:00). Natural photoperiod was maintained, with water temperature controlled at 25°C. Ammonia nitrogen levels were maintained below 0.05 mg/L, and dissolved oxygen levels were maintained above 80%.

### 2.2. Acute Toxicity Bioassay

At the commencement of the experiment, 270 healthy yellow catfish (initial weight: 12.5 ± 0.5 g) of uniform size were selected and randomly assigned to three groups, with three replicates per group. Each replicate contained 30 fish, which were reared in 300 L aquaculture tanks. Three ammonia nitrogen (T-AN) concentration gradients were established: 0 mg/L (control group, CON), 25 mg/L (low ammonia group, LOA, representing 1/10 of the 96-h lethal concentration [LC_50]_), and 125 mg/L (high ammonia group, HIA, representing 1/2 of the 96 h LC_50_) [23]. During the experiment, ammonia nitrogen concentrations were measured every 8 h using a ProQuatro Portable Multiparameter Water Quality Meter (YSI, USA) and adjusted to maintain target levels. Furthermore, natural light was maintained, and the water temperature was kept constant at 25°C, with dissolved oxygen saturation >80%. The experimental duration was 96 h.

### 2.3. Ammonia Exposure and Sampling

Following the initiation of the experiment, blood samples were collected from three randomly selected fish per tank at 6, 12, 24, 48, 72, and 96 h. Fish were anesthetized using MS-222, and blood was drawn from the caudal vein using a 1 mL syringe. Blood samples were centrifuged at 4°C and 3000 r/min for 10 min to separate serum, which was then stored at −20°C for ammonia concentration analysis. At the conclusion of the experiment, serum and liver tissue were collected from five randomly selected fish per tank. Liver samples were processed as follows: a portion was fixed in a tissue fixative for subsequent periodic acid-Schiff stain (PAS) staining and Oil Red O staining analysis. Another portion was stored at −20°C for glycogen, total cholesterol (TC) and total triglyceride (TG) content determination. The final portion was flash-frozen in liquid nitrogen and stored at −80°C for transcriptomic sequencing, metabolomics analysis, and real-time quantitative PCR (qPCR) detection.

### 2.4. Histopathological Observation

Three yellow catfish liver samples per group were fixed in 4% formaldehyde for 24 h, paraffin-embedded, sectioned, deparaffinized, rehydrated, and stained with Schiff's reagent. Sections were observed under a 400x Nikon ECLIPSE E100 microscope, with images captured via HTC5.0 CCD and WT1000GM software. Additional samples fixed in 4% paraformaldehyde overnight at 4°C were stained with Oil Red O after PBS washes and dehydration in 60% isopropanol, followed by 3 h dark incubation. Poststaining, samples were washed, dehydrated, and sealed with glycerin for microscopic observation and ImageJ-based quantification.

### 2.5. Determination of Biochemical Index

The serum ammonia (A086-1-1), glucose (F006-1-1), and liver total TGs (A110-1-1), TC (A111-1-1), and glycogen (A043-1-1) levels were quantified using commercially available kits from Nanjing Jiancheng Bioengineering Institute Co., Ltd. The assays were performed strictly in accordance with the manufacturer's instructions. Absorbance measurements were conducted using a PT-3502C microplate reader from Beijing Putian New Bridge Technology Co., Ltd. The concentrations of each biochemical parameter were subsequently calculated based on the formulas provided in the kit protocols.

### 2.6. Transcriptomics Analysis

Liver samples of yellow catfish treated for 96 h in CON and HIA groups were collected, and three biological replicates were randomly selected from each group. Total RNA was extracted from each sample using Trizol reagent (Invitrogen, USA) according to the instructions. Total RNA concentration was determined using a NanoDrop spectrophotometer (Thermo Scientific, USA), and its integrity was assessed by electrophoresis on a 1% agarose gel. An equal amount of total RNA from each sample was used to enrich eukaryotic mRNA using Oligo (dT) magnetic beads. The enriched mRNAs were randomly interrupted by adding fragmentation buffer. Then, the first strand cDNA was synthesized from the interrupted mRNA using random hexamers. Reaction buffer, dNTPs, and DNA polymerase I were then added to synthesize the second strand of cDNA. The resulting double-stranded cDNA was purified by AMPure XP beads (Beckman Coulter, USA). The purified double-stranded cDNA was ligated by end repair, A-tail addition and sequencing adapter in turn. Ligation products were again subjected to fragment size selection using AMPure XP beads and finally enriched by PCR amplification to construct the final cDNA library. After the quality of the library was qualified by an Agilent 2100 bioanalyzer (Agilent Technologies, USA), APPLIED PROTEIN TECHNOLOGY Co., Ltd. (Shanghai, China) was commissioned to perform double-end sequencing on an Illumina HiSeq 2000 platform. Significant differential expression is indicated when |log_2_^FC^|>1 and *p* < 0.05.

### 2.7. Untargeted Metabolomics Analysis

Liver tissue samples from yellow catfish subjected to 96 h treatments in CON and HIA groups were collected, with three biological replicates randomly selected from each group. Liver tissues stored at −80°C were transferred to 4°C for thawing. Following thawing, 0.2 g of tissue was precisely weighed into a precooled 2 mL centrifuge tube, and 1 mL of precooled methanol/acetonitrile/water extraction solvent (2:2:1, v/v/v) was added. The samples were ultrasonically homogenized under ice bath conditions for 30 min, then incubated at −20°C for 10 min. Subsequently, samples were centrifuged at 14,000 × *g* for 20 min at 4°C. The supernatant was collected and subjected to vacuum lyophilization. Prior to mass spectrometric analysis, the dried extracts were reconstituted in 100 μL of acetonitrile/water (1:1, v/v), vortexed thoroughly, then centrifuged at 14,000 × *g* for 15 min at 4°C. The supernatant was transferred to injection vials for LC–MS analysis. The metabolomic profiling was conducted by Shanghai APPLIED PROTEIN TECHNOLOGY Co., Ltd. (Shanghai, China). Raw MS data were converted to mzXML format using the MSConvert tool within the ProteoWizard software suite. The converted files were imported into XCMS for peak detection, alignment, and quantification. Metabolite identification was performed by matching the measured exact mass and MS/MS spectra against an internal standard database. Differential metabolites were identified using the online platform MetaboAnalyst (https://www.metaboanalyst.ca/), with screening criteria set as fold change >1.0 or <−1.0 and *p*-value < 0.05. KEGG pathway enrichment analysis was also conducted via MetaboAnalyst, with a significance threshold of *p* < 0.05.

### 2.8. RNA Extraction and qPCR Analysis

The concentration and purity of RNA were quantified using a ThermoNanoDrop 2000/2000C spectrophotometer. RNA integrity was assessed via 1% agarose gel electrophoresis. The RNA stock solution was stored at −80°C. For cDNA synthesis, the TransScript One-Step gDNA Removal and cDNA synthesis kit from TransGen Biotech (Beijing, China) was employed. cDNA was synthesized using the SuperMix protocol with total RNA as the template and stored at −20°C until further analysis. qPCR was conducted on an Applied Biosystems StepOnePlus system utilizing the Green qPCR Master Mix kit, with cDNA derived from reverse transcription serving as the template. The reaction mixture (20 μL) comprised 10.0 μL of Master Mix, 6.4 μL of RNase-free ddH_2_O, 0.8 μL each of forward and reverse primers, and 2.0 μL of cDNA template. The thermal cycling conditions included an initial denaturation at 95°C for 60 s, followed by 40 cycles of 95°C for 10 s and 60°C for 30 s. The *β-actin* and *EF1*α gene served as the internal control, and relative gene expression levels were calculated using the 2^−ΔΔCt^ method. All primers were synthesized by Sangon Biotech (Shanghai) Co., Ltd. (Shanghai, China), with sequences listed in Table 1.

### 2.9. Statistical Analysis

Experimental data are expressed as mean ± standard error (SEM). Independent samples *t*-test (for two-group comparisons) or one-way ANOVA (for three or more groups) are performed using SPSS 19.0 software (IBM, USA). Duncan's multiple range test is applied for post hoc analysis of intergroup differences when significant differences are detected; *p* < 0.05 is considered statistically significant.

## 3. Results

### 3.1. Effect of Acute AS on Serum Ammonia Level

**Figure 1:**
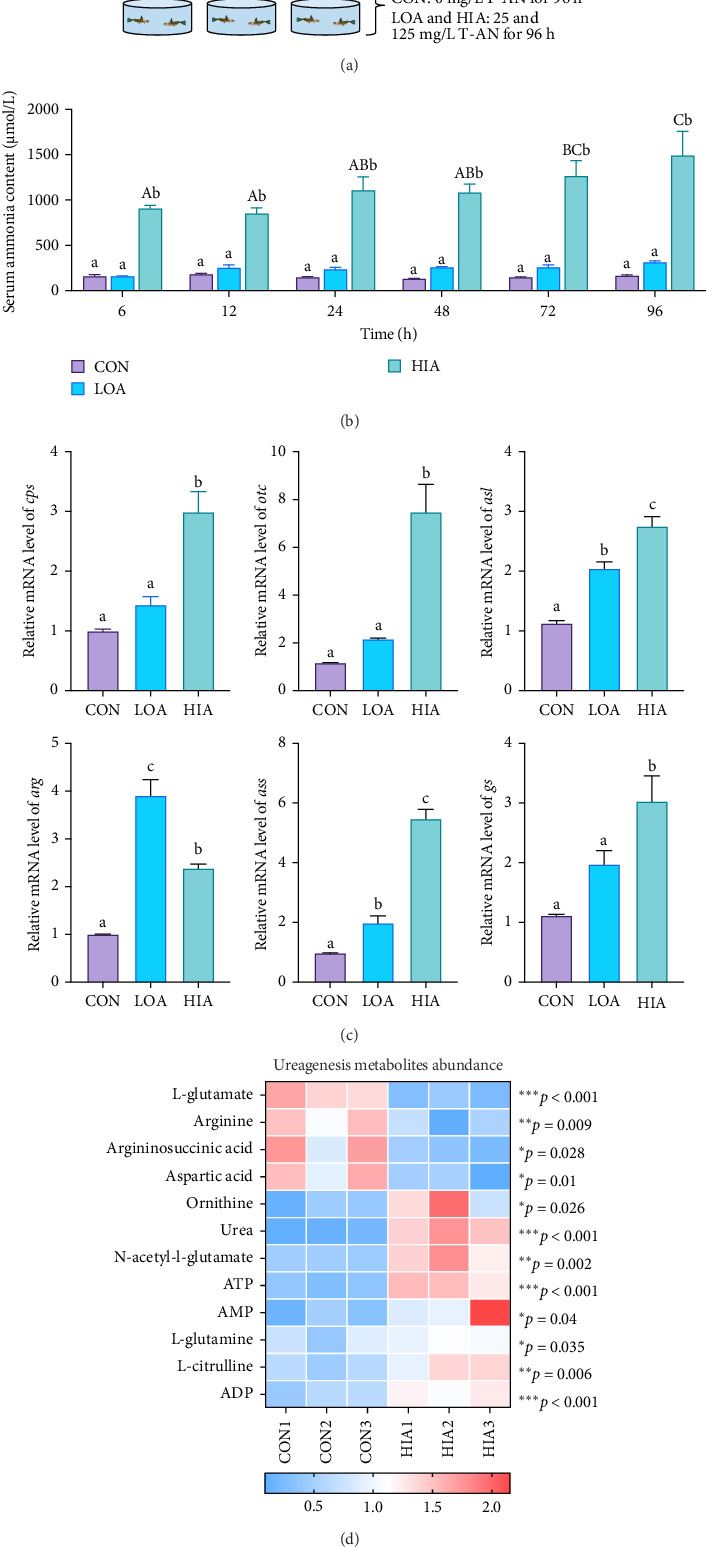
(A) Experimental design diagram. (B) Contents of ammonia in the serum of yellow catfish under ammonia stress. Different capital letters “A”, “B”, and “C” indicate significant differences in the same set of data at different time points. The capital “C” represents larger values. Different lowercase letters “a”, “b”, and “c represent significant differences between groups at the same time point. The lowercase letter “c” represents larger values. (C) The expression level of genes related to urea synthesis. Different letters “a”, “b”, and “c indicate significant differences between CON, LOA, and HIA groups. (D) The contents of metabolites related to urea synthesis under ammonia stress. *p* < 0.05 is considered statistically significant. An asterisk (*⁣*^*∗*^) indicates *p* < 0.05, two asterisks (*⁣*^*∗∗*^) denote *p* < 0.01, and three asterisks (*⁣*^*∗∗∗*^) represent *p* < 0.001.

Figure 1A briefly depicts the experimental design. Figure 1B illustrates the changes in blood ammonia in yellow catfish during 96 h of AS. There was no significant change in serum ammonia level between the CON group and the LOA group within 96 h (*p* > 0.05). The serum ammonia level in the HIA group began to increase at 24 h, this trend increased significantly at 72 h and reached the highest level at 96 h (*p* < 0.05). Longitudinally, there was no significant difference in serum ammonia level between the LOA group and the CON group at 6, 12, 24, 48, 72, and 96 h (*p* > 0.05). The serum ammonia level in the HIA group was significantly higher than that in CON and LOA groups (*p* < 0.05).

### 3.2. Effects of Acute AS on Genes and Metabolites Related to Ammonia Detoxification

**Figure 2:**
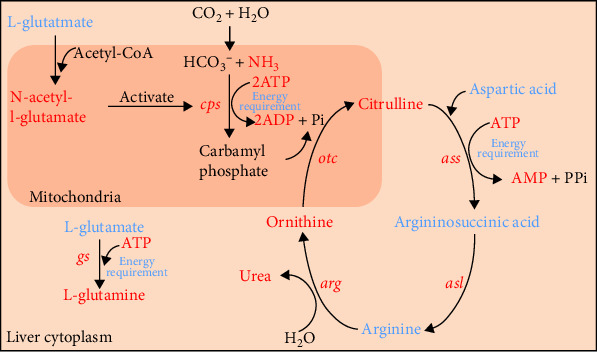
Coexpression profile of genes and metabolites involved in ureagenesis. Blue indicates decreased metabolites and red indicates upregulated metabolites and genes.

As shown in Figure 1C,D, following 96 h of AS, the liver mRNA expression levels of the ureagenesis and glutamine synthetase in the HIA group were significantly elevated compared to the CON and LOA groups (*p* < 0.05). The LOA group exhibited significantly increased mRNA levels of argininosuccinic acid lyase (*asl*), argininosuccinate synthetase (*ass*), and arginase (*arg*) relative to the CON group (*p* < 0.05). No significant differences were observed in the mRNA expression of carbamoyl-phosphate synthase (*cps*), ornithine carbamoyltransferase (*otc*), and glutamine synthetase (*gs*) between the LOA and CON groups (*p* > 0.05). In the HIA group, the concentrations of glutamate, arginine, argininosuccinic acid, and aspartic acid involved in the ammonia detoxification pathway were significantly decreased compared to the CON group (*p* < 0.05), whereas ornithine, urea, N-acetylglutamate, ATP, AMP, glutamine, citrulline, and ADP levels were significantly increased (*p* < 0.05). A schematic diagram illustrating the coexpression of ammonia detoxification-related genes and metabolites is presented in Figure 2.

### 3.3. Effects of Acute AS on Physiological States

**Figure 3:**
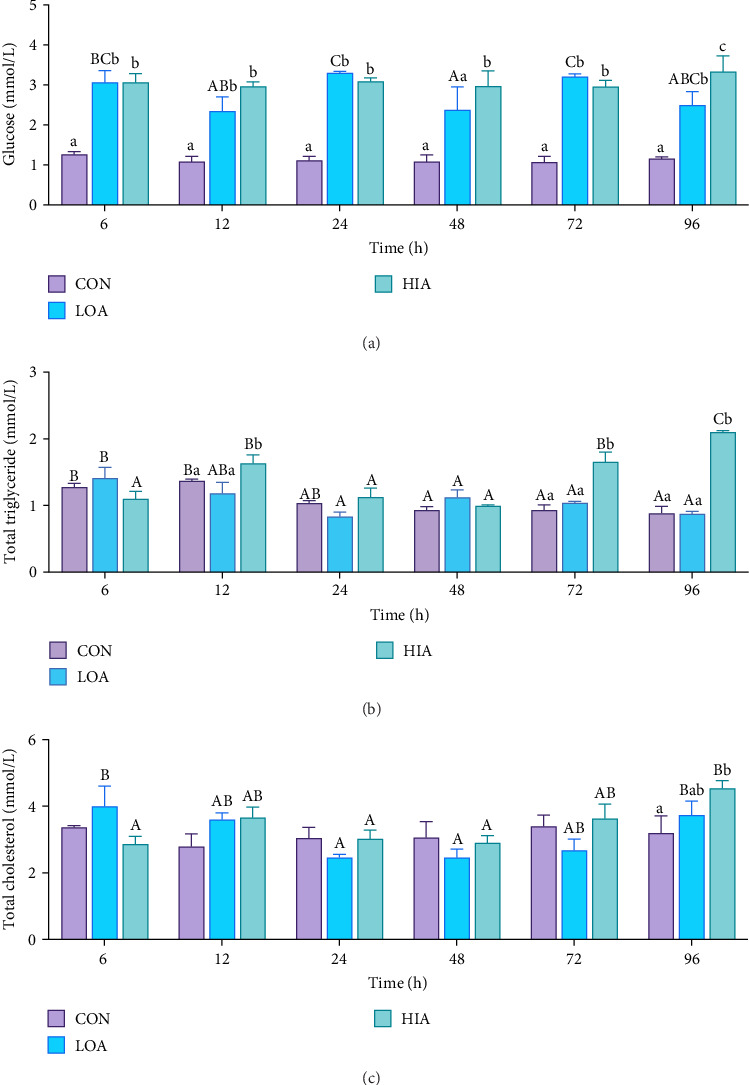
(A) Contents of glucose in the serum of yellow catfish under ammonia stress. Contents of (B) total triglycerides and (C) total cholesterol in the liver of yellow catfish under ammonia stress. At the same time point, different lowercase letters “a”, “b,” and “c” indicate significant differences between the data of each group. The lowercase letter “c” represents larger values. Different capital letters “A”, “B,” and “C” indicate significant differences in the same set of data at different time points. The capital “C” represents larger values.

As shown in Figure 3A, within the same group at different time points, serum glucose levels in the CON group showed no significant variation (*p* > 0.05). The LOA group exhibited peak serum glucose concentrations at 24 and 72 h, with the lowest level at 48 h (*p* < 0.05). The HIA group's serum glucose levels remained unchanged across different time points (*p* > 0.05). At the same time point, serum glucose concentrations in the LOA and HIA groups were significantly higher than those in the CON group (*p* < 0.05). As depicted in Figure 3B, within the same group at different time points, total TG levels in the CON group significantly decreased at 48, 72, and 96 h compared to 6, 12, and 24 h (*p* < 0.05). In the LOA group, total TG levels significantly declined at 24, 48, 72, and 96 h relative to 6 and 12 h (*p* < 0.05). Conversely, the HIA group showed significant increases in total TG levels at 12, 72, and 96 h compared to 6 and 24 h (*p* < 0.05). Between groups at the same time points, the HIA group's total TG levels were significantly higher at 12, 72, and 96 h compared to the CON and LOA groups (*p* < 0.05). As illustrated in Figure 3C, within the same group at different time points, the LOA group's TC levels were significantly lower at 24 and 48 h compared to 6 and 96 h (*p* < 0.05). The HIA group's TC levels were significantly lower at 6, 24, and 48 h compared to 96 h (*p* < 0.05). At the same time point, the HIA group's TC was significantly elevated at 96 h compared to the CON group (*p* < 0.05).

### 3.4. Effects of Acute AS on Glycogen Levels and Lipid Droplet Accumulation in the Liver

**Figure 4:**
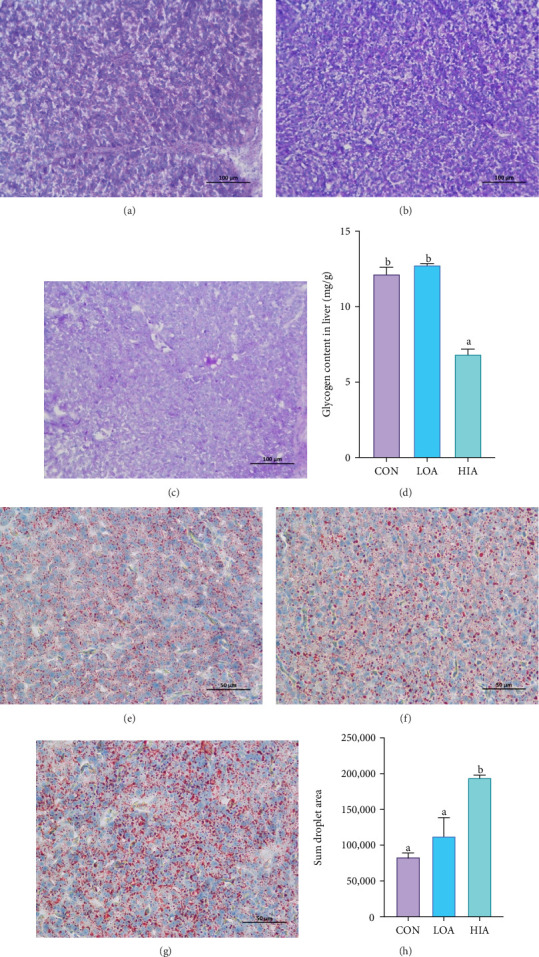
Periodic acid-schiff staining of glycogen in the liver tissues of the ammonium stress groups: (A) CON group, (B) LOA group, and (C) HIA group. (D) Glycogen content in the liver. (E) CON group (F) LOA (G) HIA group Oil Red O staining of lipid droplets in liver tissue. (H) The sum areas of ORO staining were quantified using ImageJ software. Different lowercase letters “a, b, c” indicate statistically significant differences among the data groups. The lowercase letter “b” denotes a higher value.

As shown in Figure 4A–C, the PAS-stained area gradually diminishes with increasing AS concentration. Figure 4D indicates that after 96 h of ammonia exposure, liver glycogen content in the HIA group is significantly reduced compared to the CON and LOA groups (*p* < 0.05), with no significant difference between the CON and LOA groups (*p* > 0.05). Additionally, the number of liver lipid droplets in the HIA group is significantly elevated relative to the CON and LOA groups (*p* < 0.05), while there is no significant difference in lipid droplet count between the CON and LOA groups (*p* > 0.05).

### 3.5. Effect of Acute AS on Liver Transcriptome

**Figure 5:**
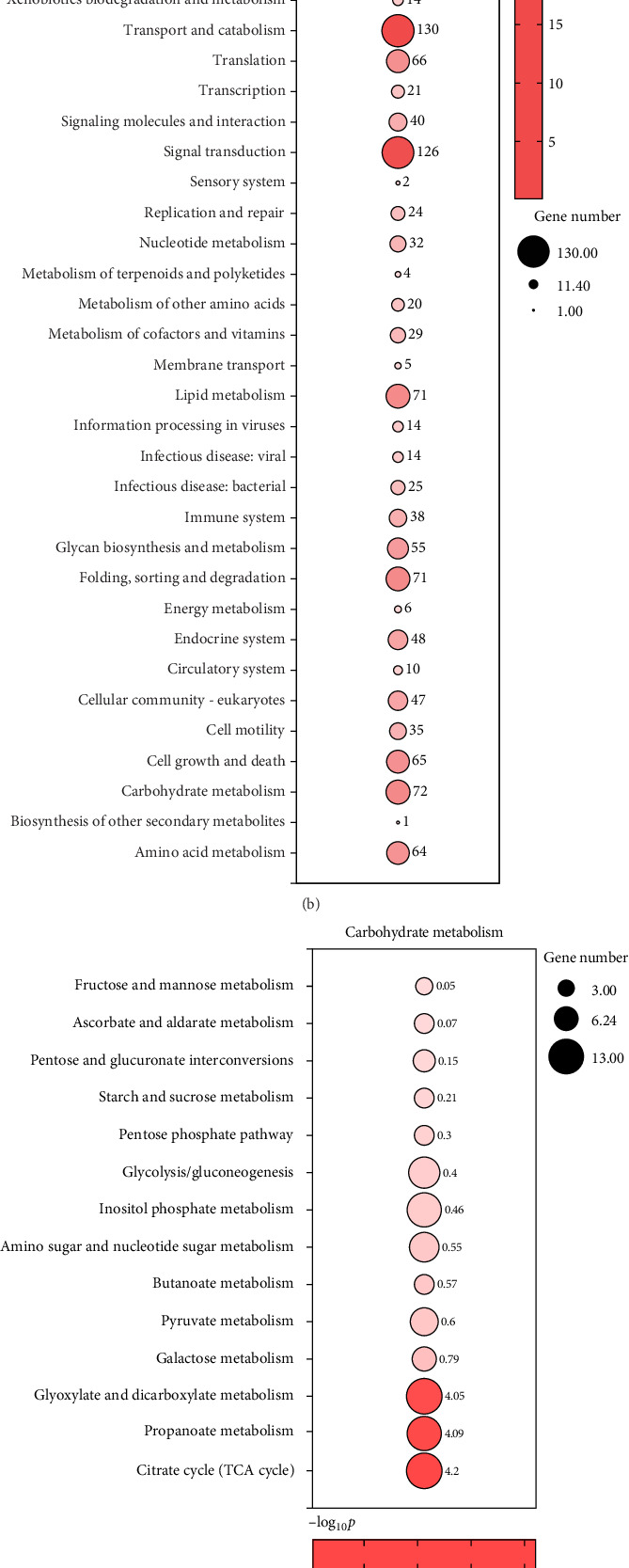
Transcriptomic analysis of yellow catfish liver: (A) Heatmap of differentially expressed genes. (B) Functional enrichment analysis of differentially expressed genes in level 2. (C) Signal transduction pathways associated with carbohydrate metabolism. (D) Signal transduction pathways related to lipid metabolism. Larger circles indicate a greater number of genes involved in the pathway. Redder circles signify higher enrichment significance.

**Figure 6:**
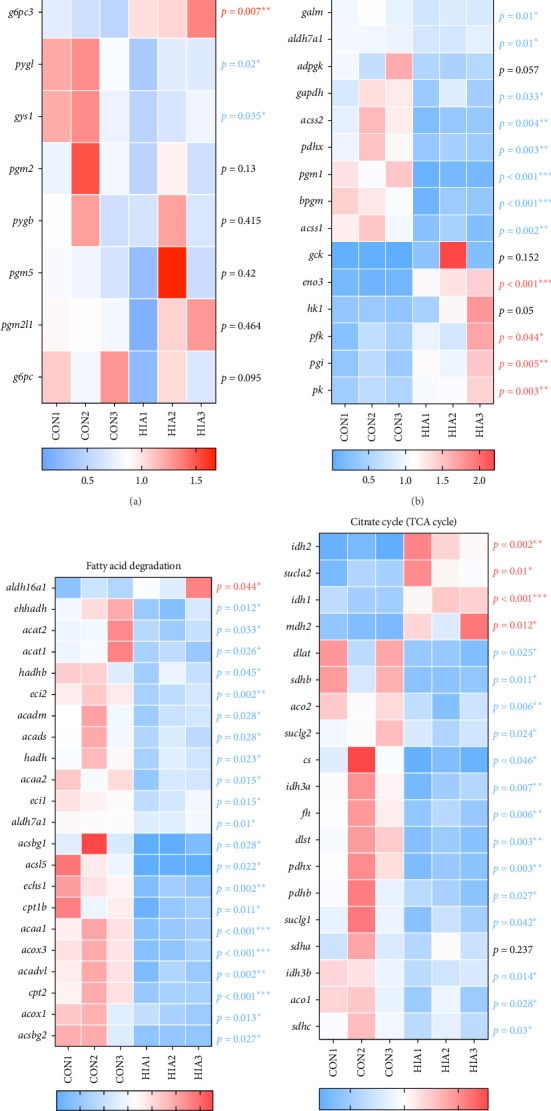
Heatmap expression profiles of genes involved in the (A) starch and sucrose metabolism, (B) glycolysis, (C) fatty acid degradation, (D) TCA cycle (*n* = 3). *p* < 0.05 is considered statistically significant. An asterisk (*⁣*^*∗*^) indicates *p* < 0.05, two asterisks (*⁣*^*∗∗*^) denote *p* < 0.01, and three asterisks (*⁣*^*∗∗∗*^) represent *p* < 0.001.

Due to the significant impact of AS on carbohydrate and lipid metabolism in the liver of yellow catfish, we conducted transcriptomic and metabolomic analyses on the CON and HIA groups. As shown in Figure 5A, after 96 h of AS, transcriptomic results revealed a total of 2751 differentially expressed genes, with 386 significantly upregulated and 2365 significantly downregulated genes. Figure 5B illustrates KEGG pathway enrichment analysis of these differentially expressed genes, highlighting pathways such as “transport and catabolism,” “signal transduction,” “lipid metabolism,” “protein folding, sorting, degradation,” and “carbohydrate metabolism.” As depicted in Figure 5C, pathways including the “TCA cycle,” “propanoate metabolism,” and “glyoxylate and dicarboxylate metabolism” were notably enriched. Figure 5D indicates significant enrichment in pathways related to “fatty acid degradation,” “biosynthesis of unsaturated fatty acids,” and “glycerophospholipid metabolism.” As shown in Figure 6A, within the “starch and sucrose metabolism” pathway, the counts of *gys2*, *pgm1*, *pygl*, and *gys1* in the HIA group are significantly reduced compared to the control group, while the count of *g6pc3* is significantly elevated (*p* < 0.05). However, the counts of *pgm2*, *pgb*, *pgm5*, *pgm2/1*, and *g6pc* do not reach statistical significance (*p* > 0.05). As depicted in Figure 6B, in the “glycolysis/gluconeogenesis” pathway, the counts of *pgk1*, *dlat*, *galm*, *aldh7a1*, *gapdh*, *acss2*, *pdhx*, *pam1*, and *acss1* are significantly downregulated in the HIA group compared to controls. Conversely, the counts of *eno3*, *pfk*, *pgi*, and *pk* are significantly upregulated (*p* < 0.05). Figure 6C illustrates that in the “fatty acid degradation” pathway, the counts of *ehhadh*, *acat2*, *acat1*, *hadhb*, *eci2*, *acadm*, *acads*, *hadh*, *acaa2*, *eci1*, *aldh7a1*, *acsbg1*, *acsl5*, echs1, *cpt1b*, *acaa1*, *acox3*, *acadvl*, *cpt2*, *acox1*, and *acsbg2* are significantly decreased in the HIA group relative to controls (*p* < 0.05). Conversely, the count of aldh16a1 is significantly increased (*p* < 0.05). Figure 6D demonstrates that in the “TCA cycle”, the counts of *sucla2*, *idh2*, and *mdh2* are significantly elevated in the HIA group compared to controls (*p* < 0.05). In contrast, the counts of *dlat*, *sdhb*, *aco2*, *suclg2*, *cs*, *idh3a*, *fh*, *dlst*, *pdhx*, *pdhb*, *suclg1*, *idh3b*, *aco1*, *sdhc* are significantly decreased (*p* < 0.05).

### 3.6. Effects of Acute AS on Liver Metabolome

**Figure 7:**
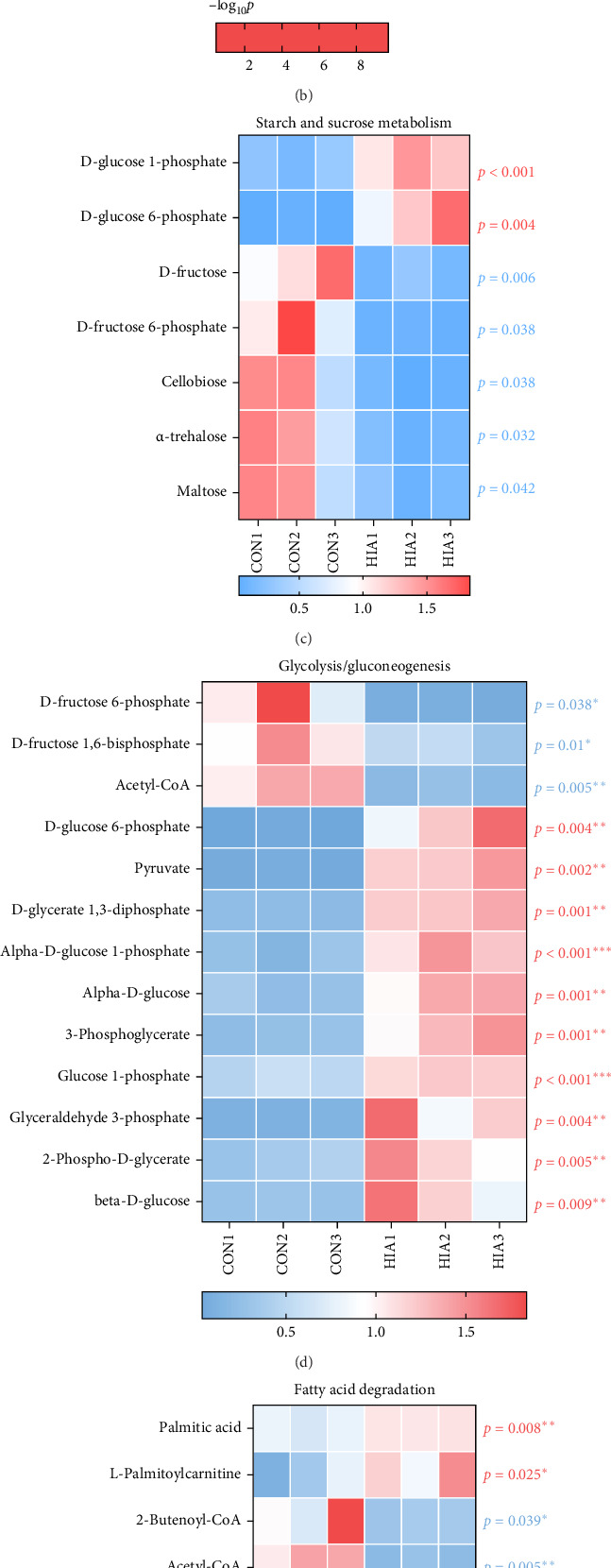
Metabolomic analysis of yellow catfish liver. (A) Scatter plot of differential metabolites, with red dots indicating significantly upregulated metabolites and blue dots indicating significantly downregulated metabolites. (B) Functional enrichment analysis of differential metabolites, where larger circles represent pathways with a higher number of metabolites, and redder circles indicate greater enrichment significance. The heatmap expression profiles of (C) starch and sucrose metabolism, (D) glycolysis, (E) fatty acid degradation, and (F) TCA cycle (*n* = 3). *p* < 0.05 is considered statistically significant. An asterisk (*⁣*^*∗*^) indicates *p* < 0.05, two asterisks (*⁣*^*∗∗*^) denote *p* < 0.01, and three asterisks (*⁣*^*∗∗∗*^) represent *p* < 0.001.

As shown in Figure 7A,B, after 96 h of AS, metabolomic analysis of the HIA and CON groups identified a total of 293 differential metabolites, with 212 significantly upregulated and 81 significantly downregulated. KEGG pathway enrichment analysis revealed that the “TCA cycle,” “butanoate metabolism,” “glycolysis or gluconeogenesis,” “galactose metabolism,” “fructose metabolism,” and “glyoxylate and dicarboxylate metabolism” were notably affected. As depicted in Figure 7C, within the “starch and sucrose metabolism” pathway, the levels of D-glucose 1-phosphate and D-glucose 6-phosphate were significantly elevated in the HIA group compared to the CON group (*p* < 0.05), while D-fructose, D-fructose 6-phosphate, cellobiose, alpha-trehalose, and maltose were significantly decreased (*p* < 0.05). Figure 7D illustrates that in the “glycolysis/gluconeogenesis” pathway, the concentrations of D-fructose 6-phosphate, D-fructose 1,6-bisphosphate, and acetyl-CoA were significantly reduced in the HIA group relative to the CON group (*p* < 0.05). Conversely, levels of D-glucose 6-phosphate, pyruvate, D-glycerate 1,3-diphosphate, alpha-D-glucose 1-phosphate, alpha-D-gucose, 3-phosphoglycerate, glucose 1-phosphate, glyceraldehyde 3-phosphate, 2-phospho-D-glycerate, and beta-D-glucose were significantly increased (*p* < 0.05). As shown in Figure 7E, during “fatty acid catabolism,” the concentrations of palmitic acid and L-palmitoylcarnitine were significantly higher in the HIA group compared to the CON group (*p* < 0.05). Conversely, levels of palmitic acid, L-palmitoylcarnitine, 2-butenoyl-CoA, acetyl-CoA, palmitoyl-CoA, (S)-3-hydroxybutanoyl-CoA, butyryl-CoA, and acetoacetyl-CoA were significantly decreased (*p* < 0.05). Figure 7F demonstrates that in the “TCA cycle,” the levels of oxaloacetate, succinyl-CoA, malate, citrate, alpha-ketoglutaric acid, NADH, and fumarate were significantly reduced in the HIA group compared to the CON group (*p* < 0.05). Conversely, Cis-aconitate, NAD^+^, AMP, isocitrate, ADP, succinate, ATP, and oxalosuccinate levels were significantly elevated (*p* < 0.05).

### 3.7. Expression Profiling of Genes Involved in Carbohydrate and Lipid Metabolism

**Figure 8:**
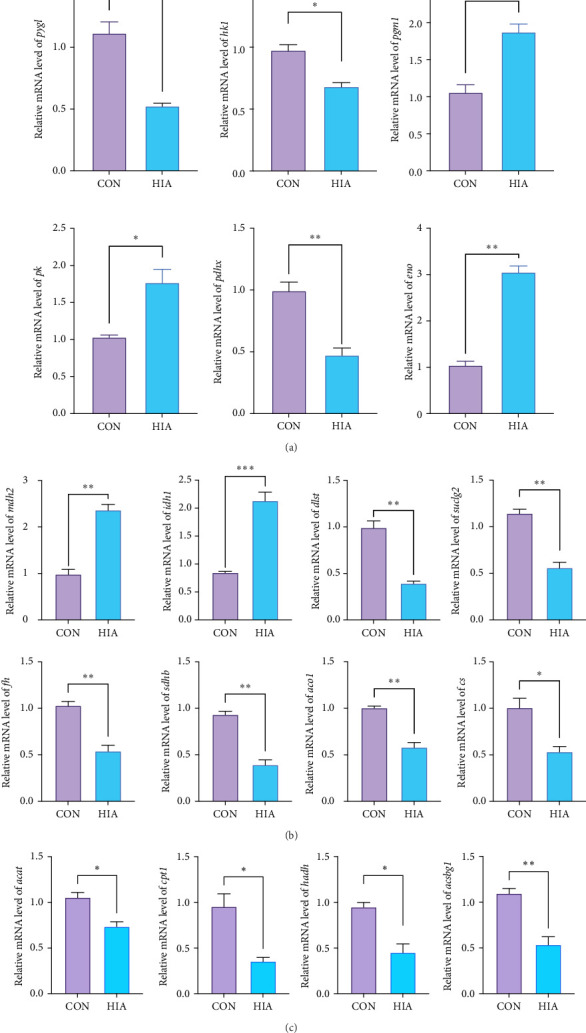
Quantitative PCR analysis of key genes involved in (A) glycolysis, (B) TCA cycle, and (C) fatty acid catabolism (*n* = 3). *p* < 0.05 is considered statistically significant. An asterisk (*⁣*^*∗*^) indicates *p* < 0.05, two asterisks (*⁣*^*∗∗*^) denote *p* < 0.01, and three asterisks (*⁣*^*∗∗∗*^) represent *p* < 0.001.

**Figure 9:**
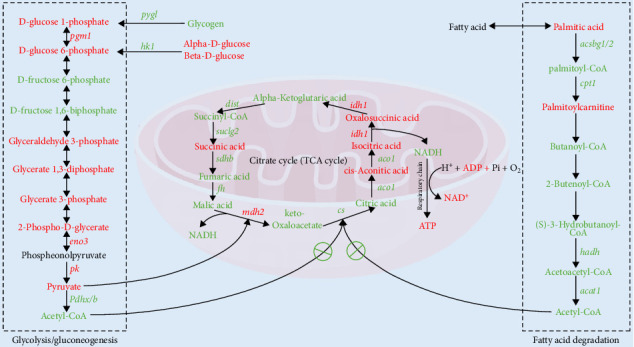
The objective of the transcriptomic and metabolomic analysis of yellow catfish liver. The red words indicate upregulated genes and metabolites, while the green words represent downregulated genes and metabolites.

As shown in Figure 8, after 96 h of AS, the relative gene expression levels of glycolysis-related genes *pygl*, *hk1*, and *pdhx* in the HIA group were significantly decreased compared to the CON group (*p* < 0.05), while the expression levels of *pgm1*, *pk*, and *eno* were significantly increased (*p* < 0.05). In the HIA group, genes involved in the TCA cycle, *mdh2*, and *idh1*, showed significantly elevated expression levels compared to the CON group, whereas *dlst*, *suclg2*, *fh*, *sdhb*, *aco1*, and *cs* exhibited significantly reduced expression (*p* < 0.05). Additionally, genes associated with fatty acid catabolism, *acat*, *cpt1*, *hadh*, and *acsbg1*, were significantly downregulated in the HIA group relative to the CON group (*p* < 0.05). The schematic diagram illustrating the coexpression of carbohydrate and lipid metabolism-related genes and metabolites in the liver of yellow catfish under AS is presented in Figure 9.

## 4. Discussions

Ammonia is a significant environmental stressor that limits the healthy development of aquaculture. The photosynthesis and respiration of microorganisms and algae in aquatic environments directly affect the carbon dioxide content in the water, which leads to pH fluctuations. While the concentration of NH_3_ increases with rising pH, NH_3_ accounts for only 1%–2% of the total ammonia content in the neutral pH range. However, under extreme conditions such as a pH of 9 and a water temperature of 35°C, the proportion of NH_3_ in the water can increase to 44%–53% within several hours, resulting in significant toxicity to aquatic organism [24].

This study employed histomorphology observation, physiological, and biochemical indicator assessment, and multiomics analysis to examine the impacts of acute AS on yellow catfish, with the aim of establishing a theoretical framework for understanding the response mechanisms of cultured fish to AS. Results indicated a significant increase in serum ammonia levels in yellow catfish with escalating ammonia concentrations and prolonged exposure, demonstrating dose-dependent characteristics. This suggests that environmental ammonia can enter the circulatory system via pathways such as trans-branchial epithelium transport [25], leading to systemic accumulation [26]. Ammonia accumulation in fish elicits a range of toxicological effects, encompassing oxidative stress, immune suppression, and hypoxia-induced mortality [27]. Over evolutionary timescales, the yellow catfish has developed sophisticated physiological mechanisms for ammonia detoxification. These include transmembrane ammonia efflux facilitated by ion transporters in gill epithelial cells [28], and a liver-centric metabolic detoxification pathway. This pathway involves the glutamine synthetase-mediated conjugation of ammonia and glutamate to form glutamine, as well as the conversion of ammonia to urea [29]. The findings of this investigation demonstrated that AS notably upregulated the transcription of genes associated with the ureagenesis and glutamine synthesis pathway within the liver of yellow catfish. This upregulation was concurrent with elevated glutamine and urea concentrations in the tissues, indicating a significant activation of the liver ammonia detoxification metabolic pathway. Subsequent metabolomics analysis revealed a dose-dependent reduction in liver glutamate, argininosuccinic acid, and arginine levels under AS, suggesting substrate depletion due to their involvement in glutamine synthesis and ureagenesis [25, 30]. Notably, ammonia detoxification is an energetically intensive process, critically reliant on a continuous ATP supply [31, 32]. This investigation reveals that AS administration prompts a concurrent increase in liver ATP and its catabolites, ADP and AMP, signifying a significant acceleration of intracellular nucleotide turnover to fulfill the elevated energetic requirements of detoxification. In conclusion, AS induces metabolic reprograming of liver energy pathways in yellow catfish, characterized by a strategic redistribution of energy resources to optimize ammonia clearance.

In fish energy metabolism, carbohydrates, and lipids are the primary ATP sources, with protein oxidation being less efficient [33]. Carbohydrates oxidation is faster than lipid and protein oxidation [13], but carbohydrates utilization is less efficient [34]. Therefore, aquatic animals often regulate carbohydrates and lipid metabolism concurrently to address energy deficits from environmental stress [35]. In this investigation, we aimed to elucidate the impact of AS on liver carbohydrates and lipid metabolism in yellow catfish. The findings indicated that liver glycogen reserves diminished proportionally with escalating levels of AS, while serum glucose concentrations and liver lipid droplet accumulation significantly increased. These data imply that under AS conditions, the yellow catfish liver may mobilize glycogenolysis to generate glucose for ammonia detoxification. However, since lipolysis is an energy-consuming process, suppression of lipid catabolism can conserve metabolic energy, thereby indirectly facilitating the energy requirements for ammonia detoxification. Prior research has demonstrated that Asian seabass (*Lates calcarifer*) utilizes glucose to meet energy demands during hypoxic stress induced by crowding [36], and that *Carassius auratus*, characterized by high muscle glycogen levels, exhibits enhanced ammonia detoxification capacity [37]. Concurrently, studies have indicated that AS can inhibit lipid metabolism in largemouth bass (*Micropterus salmoides*), thereby activating oxidative phosphorylation, promoting preferential carbohydrates utilization for energy, and downregulating lipid metabolism. This may represent an adaptive energy allocation strategy in response to environmental stress [21]. In summary, AS modulates the bioenergetic flux in yellow catfish, with augmented liver glycogenolysis and suppressed lipid catabolism potentially forming a novel metabolic pathway that sustains ammonia detoxification during energy-deficient states.

To validate the conclusion, transcriptome, metabolome, and qPCR analyses were integrated to investigate the mechanism of nutrient metabolism in the liver of yellow catfish under AS. Results indicated that AS suppressed the transcriptional levels of most glycolytic pathway genes. The qPCR revealed that AS inhibited liver *hk1* and *pdhx* expression, significantly increasing α-D-glucose, beta-D-glucose, and pyruvate content. This suggests that glycolysis-mediated glucose production of acetyl CoA is inhibited in the liver. Glycolysis, the initial stage of carbohydrate aerobic oxidation [38], is inhibited by ammonia, potentially impacting the subsequent tricarboxylic acid (TCA) cycle. The results of this study showed that the gene expression level of citrate synthase (*cs*) and citrate content were significantly downregulated under AS. The *cs* catalyzes the synthesis of citrate from acetyl CoA and oxaloacetate and is the initiation step of the TCA cycle [39]. Downregulation of *cs* expression directly resulted in a significant reduction in TCA cycle flux [21]. Previous studies have shown that, AS can inhibit the process of TCA cycle in gill tissues of *Litopenaeus vannamei* [40] and liver of largemouth bass (*Micropterus salmoides*) [21]. AS may inhibit *cs* gene expression, decreasing TCA cycle flux and inhibiting ATP production from glucose via glycolysis/TCA cycle. Therefore, a key characteristic of the novel bioenergetic model modulated by AS is the metabolic shift of the primary energy substrate from glucose to glycogen.

Recent investigations have demonstrated that the upregulation of lipid metabolism can markedly enhance fish tolerance to AS [41]. The present investigation revealed that AS notably downregulated the liver transcription of crucial genes involved in fatty acid catabolism (*acsbg1*, *cpt1*, *hadh*, and *acat1*). This resulted in the accumulation of palmitic acid and palmitoyl–carnitine, alongside the depletion of palmitoyl-CoA, butanoyl-CoA, and acetyl-CoA. These findings suggest that AS may impede *cpt1*-mediated mitochondrial fatty acid transport and the activity of β-oxidation enzymes, thereby disrupting the breakdown of fatty acids into acetyl-CoA and its subsequent energy provision to the TCA cycle, ultimately leading to a disruption of liver lipid metabolism. These observations align with prior studies demonstrating the inhibition of fatty acid β-oxidation in the gill tissue of juvenile four-finger threadfin (*Eleutheronema tetradactylum*) [19] and the liver of grass carp (*Ctenopharyngodon idella*) under AS [42]. Therefore, the second characteristic of this novel energy metabolic reprograming induced by AS the suppression of energetically costly lipid catabolism serves to conserve cellular energy resources.

Notably, liver tissue-based metabolomics analysis revealed a significant increase in liver ATP content under AS. This observation implies that the organism may initiate nonclassical ATP generation pathways via compensatory metabolic remodeling to preserve cellular energy homeostasis during ammonia exposure. Furthermore, our investigation demonstrated that *pygl* gene expression was significantly downregulated, and liver glycogen content decreased under AS, whereas D-glucose 1-phosphate content exhibited a significant increase. These findings suggest that AS induces substrate depletion by promoting glycogen catabolism, consequently suppressing *pygl* expression. Integrated metabolomics and transcriptomics analyses revealed significant upregulation of D-glucose 6-phosphate, glyceraldehyde 3-phosphate, pyruvate, and the expression of *eno3* and *pk*, indicating the conversion of glycogen to pyruvate via glycolysis. Further investigation revealed significant upregulation of *mdh2* and *idh1*, respectively facilitating the conversion of malate to oxaloacetate and isocitrate to α-ketoglutarate, accompanied by NADH production, providing reducing equivalents for oxidative phosphorylation [43]. Metabolite analysis demonstrated a decreased NADH/NAD^+^ ratio and an increased ATP/ADP ratio under AS. Coupled with glycogen depletion, this study elucidates that AS promotes the conversion of glycogen to pyruvate, which directly enters the TCA cycle as the primary ATP-generating pathway, ultimately leading to compensatory depletion of glycogen. Prior research has demonstrated that goldfish (*Carassius auratus L*.) utilize glycogenolysis in the liver and muscle tissues as an adaptive metabolic strategy under AS [44]. This compensatory glycogen breakdown ultimately leads to the depletion of energy reserves.

In summary, the energy metabolism pattern under AS shifts from aerobic oxidation to anaerobic glycogen metabolism. Additionally, ammonia exposure has been shown to upregulate AMPK expression in Chinese stridle-neck turtle (*Mauremys sinensis*), thereby enhancing glycolytic flux to fulfill cellular energy requirements under stress conditions [17]. This finding contrasts with our observations and collectively indicates that interspecies variability exists in the regulatory mechanisms governing energy metabolism during ammonia challenge, potentially representing an adaptive survival strategy developed through long-term evolutionary processes.

## 5. Conclusion

This study utilizes histopathological, physiological, biochemical, and multiomics analyses to systematically investigate the toxic effects of acute ammonia exposure on yellow catfish and the underlying liver energy metabolism reprograming mechanisms. Findings indicate that AS triggers liver detoxification responses, notably activating glutamine synthesis and ureagenesis. Concurrently, liver energy metabolism undergoes significant reprograming, characterized by: (1) suppression of the glycolysis—TCA cycle pathway; (2) inhibition of fatty acid β-oxidation; and (3) upregulation of glycogenolysis as the primary energy source. Pyruvate derived from glycogenolysis enters the TCA cycle, generating NADH to support oxidative phosphorylation and sustain ATP synthesis. Consequently, both the turnover rate and content of liver ATP are elevated. This study provides a theoretical basis for understanding molecular mechanisms of ammonia toxicity in aquaculture species and proposes metabolic regulation strategies. It suggests optimizing carbohydrate sources in aquafeed according to fish energy needs to ensure glycogenolysis sustains energy under AS, maintaining metabolic balance and detoxification, thus reducing ammonia toxicity.

## Figures and Tables

**Table 1:** Primers for qPCR analysis of target genes.

Target gene	Primer sequence (5′–3′)	Size (bp)	NCBI reference sequence
*cps*	F: CCTGAAGACGGTGCTGATGA	267	XM_047814812.1
	R: TCTGTGGCGATGATGGACTC	—	—
*otc*	F: TTCTACTCACCACGCTCACT	89	XM_047815226.1
	R: GTCAGTCAGGATACATACAATCAAC	—	—
*ass*	F: AGACTCACCGAATACACCAGAC	134	XM_027136752.2
	R: CTCCGACTTCATTGAGGTAGGT	—	—
*asl*	F: ATTCACACAGCCAATGAACG	343	XM_027156981.2
	R: CACTTCCCAAAGGCAGAATG	—	—
*arg*	F: AAGAAGGTTGCTGATGCTGTT	181	XM_027167958.2
	R: CTGTTGGACTGGTTAGAGGTG	—	—
*gs*	F: ATCCGTGCCTATGACCCCAA	189	XM_027160582.2
	R: GTCACAGTTAGCACTCGGGC	—	—
*pygl*	F: AATCATAGACGGCTGCCAGG	169	XM_027175050.2
	R: GGGCATAGCCAGAACAACCT	—	—
*hk1*	F: CTGTTTGAAGGCCGCATCAC	132	XM_027174934.2
	R: CTCTACTCCCAGACGGGTCA	—	—
*pgm1*	F: GCTTACAGCGACCAAAAGCC	119	XM_027147216.2
	R: GAGGTCTCGATGGCCGAAAT	—	—
*mdh2*	F: TGCGGCTCTAAAGGGATGTG	258	XM_027158537.2
	R: TCCAGCGTTGTGACTCCAAA	—	—
*idh1*	F: CCTGCACCCTCAGATCTACC	192	XM_027143610.2
	R: AGCTGTGCAGGTCTAACTCC	—	—
*dlst*	F: TCAAGACCACTGCATCCCAC	125	XM_027172437.2
	R: CTCATCTTCTGCGACGGTGT	—	—
*suclg2*	F: AGGCAGGCTGCAGATCAAAT	203	XM_027155815.2
	R: TCTATGGGATCGCTTTCGGC	—	—
*pk*	F: TGCTGTTCTGGATGGAGCTG	231	XM_027159273.2
	R: GCACTGGCACAGCATTTGAA	—	—
*pdhx*	F: CTGGTGATGCTCTCTGCGAA	185	XM_027173431.2
	R: GACAAGCGGAGGGATTTCCA	—	—
*eno*	F: TTCGACTCACGTGGAAACCC	219	XM_047807253.1
	R: GACGGACACGTCCTGGTTAC	—	—
*fh*	F: GCTGCCTCAGGATGACAAGT	227	XM_027132780.2
	R: CTTGGTGTCCAAGCCGTAGT	—	—
*sdhb*	F: CGAGCGGGCCTCTTTATTCT	144	XM_027173961.2
	R: CCGAACCACGTTCACAAACC	—	—
*aco1*	F: TCCAGCAAGAAGACCCGAAC	213	XM_027133527.2
	R: GCGAGGCCTGGTTTAGAAGT	—	—
*cs*	F: TATTGCCAAAGGCTGCAGGA	182	XM_027144899.2
	R: AGGGTGCAAGTTGGTTGGAA	—	—
*acat*	F: CGTGGCTCCTATCGACTTCC	132	XM_027158385.2
	R: TTGGCCAGAACAACCACACT	—	—
*cpt1*	F: TTGCGCTAGCAAACAGGGTA	264	XM_027136894.2
	R: GGGCAGCCAGTTTCTCTTCT	—	—
*hadh*	F: CCCCGTCCCTATGATGAAGC	208	XM_027145784.2
	R: CTCCTTGGAACCGTGTCCTC	—	—
*acsbg1*	F: ACGAGAAGGAGCAACACAGC	135	XM_027173435.2
	R: CTGGAGCCAGAATTTCCCCTG	—	—
*β-actin*	F: GACATCCACCCAAAAGCCAA	136	XM_027148463.2
	R: CGTGCTCAATGGGGTACT	—	—
*EF1*α	F: CCCCTGGACACAGAGACTTT	253	XM_027175544.2
	R: GCGCTGACTTCTTTGGTGAT	—	—

Abbreviations:* β-actin*, beta- actin; *acsbg1*, acyl-coa synthetase bubblegum family member 1; *cpt1*, carnitine palmitoyltransferase 1; *EF1*α, elongation factor 1-alpha.; *ass*, argininosuccinate synthase; *acat*, acetyl-CoA acetyltransferase*; arg, arginase; asl, argininosuccinate lyase; cps, carbamoyl-phosphate synthase; cs, citrate synthase; dlst, dihydrolipoamide s-succinyltransferase; aco1*, acyl-coenzyme a oxidase1; *eno*, enolase 1a; *fh*, fumarate hydratase; *gs*, glutamine synthase; *hadh*, hydroxyacyl-CoA dehydrogenase; *hk1*, hexokinase 1; *idh1*, isocitrate dehydrogenase; *mdh2*, malate dehydrogenase 2; *otc*, ornithine transcarbamylase; *pdhx*, pyruvate dehydrogenase complex component x; *pgm1*, phosphoglucomutase 1; *pk*, pyruvate kinase; *pygl*, glycogen phosphorylase; *sdhb*, succinate dehydrogenase complex; *suclg2*, succinate-coa ligase gdp-forming subunit beta.

## Data Availability

The data will be made available upon request.

## Nomenclature

*acat*: Acetyl-CoA acetyltransferase
*aco1*: Acyl-coenzyme a oxidase1
*acsbg1*: Acyl-coa synthetase bubblegum family member 1
*arg*: Arginase
*asl*: Argininosuccinate lyase
*ass*: Argininosuccinate synthase
*β-actin*: Beta-actin
*cpt1*: Carnitine palmitoyltransferase 1
*cps*: Carbamoyl-phosphate synthase
*cs*: Citrate synthase
*dlst*: Dihydrolipoamide s-succinyltransferase
*EF1*α: Elongation factor 1-alpha
*eno*: Enolase 1a
*fh*: Fumarate hydratase
*gs*: Glutamine synthase
*hadh*: Hydroxyacyl-CoA dehydrogenase
*hk1*: Hexokinase 1
*idh1*: Isocitrate dehydrogenase
*mdh2*: Malate dehydrogenase 2
*otc*: Ornithine transcarbamylase
*pdhx*: Pyruvate dehydrogenase complex component x
*pgm1*: Phosphoglucomutase 1
*pk*: Pyruvate kinase
*pygl*: Glycogen phosphorylase
*sdhb*: Succinate dehydrogenase complex
*suclg2*: Succinate-coa ligase gdp-forming subunit beta.

## References

[1] Edwards T. M., Puglis H. J., Kent D. B., Duran J. L., Bradshaw L. M., Farag A. M. (2024). Ammonia and Aquatic Ecosystems–A Review of Global Sources, Biogeochemical Cycling, and Effects on Fish. *Science of the Total Environment*.

[2] Ip Y. K., Chew S. F. (2010). Ammonia Production, Excretion, Toxicity, and Defense in Fish: A Review. *Frontiers in Physiology*.

[3] Bhatti J. S., Bhatti G. K., Reddy P. H. (2017). Mitochondrial Dysfunction and Oxidative Stress in Metabolic Disorders-A Step Towards Mitochondria Based Therapeutic Strategies. *Biochimica et Biophysica Acta (BBA)-Molecular Basis of Disease*.

[4] Liu M. J., Guo H. Y., Zhu K. C. (2021). Effects of Acute Ammonia Exposure and Recovery on the Antioxidant Response and Expression of Genes in the Nrf2-Keap1 Signaling Pathway in the Juvenile Golden Pompano (*Trachinotus ovatus*). *Aquatic Toxicology*.

[5] Wang H. J., Xiao X. C., Wang H. Z. (2017). Effects of High Ammonia Concentrations on Three Cyprinid Fish: Acute and Whole-Ecosystem Chronic Tests. *Science of the Total Environment*.

[6] Sinha A. K., Matey V., Giblen T., Blust R., De Boeck G. (2014). Gill Remodeling in Three Freshwater Teleosts in Response to High Environmental Ammonia. *Aquatic Toxicology*.

[7] Farag A. M., Harper D. D., Cozzarelli I. M. (2022). Using Biological Responses to Monitor Freshwater Post-Spill Conditions Over 3 Years in Blacktail Creek, North Dakota, USA. *Archives of Environmental Contamination and Toxicology*.

[8] Egnew N., Renukdas N., Ramena Y. (2019). Physiological Insights Into Largemouth Bass (*Micropterus salmoides*) Survival during Long-Term Exposure to High Environmental Ammonia. *Aquatic Toxicology*.

[9] Rice S. D., Bailey J. E. (1980). Survival, Size, and Emergence of Pink Salmon, *Oncorhynchus gorbuscha*, Alevins After Short- and Long-Term Exposures to Ammonia. *Fishery Bulletin*.

[10] Zhang W. X., Xia S. L., Zhu J. (2019). Growth Performance, Physiological Response and Histology Changes of Juvenile Blunt Snout Bream, *Megalobrama amblycephala* Exposed to Chronic Ammonia. *Aquaculture*.

[11] Xi L. W., Lu Q. S., Liu Y. L. (2023). Study on Carbohydrate Metabolism in Adult Zebrafish (*Danio rerio*). *Aquaculture Nutrition*.

[12] Guo J. S., Fu Y. H., Wu Z. H. (2022). Effects of Dietary Carbohydrate Levels on Growth Performance, Body Composition, Glucose/Lipid Metabolism and Insulin Signaling Pathway in Abalone. *Aquaculture*.

[13] Kamalam B. S., Medale F., Panserat S. (2017). Utilisation of Dietary Carbohydrates in Farmed Fishes: New Insights on Influencing Factors, Biological Limitations and Future Strategies. *Aquaculture*.

[14] Zhang X. S., Jin M., Luo J. X. (2022). Effects of Dietary Carbohydrate Levels on the Growth and Glucose Metabolism of Juvenile Swimming Crab, *Portunus trituberculatus*. *Aquaculture Nutrition*.

[15] Boonanuntanasarn S., Jangprai A., Kumkhong S. (2018). Adaptation of Nile Tilapia (*Oreochromis niloticus*) to Different Levels of Dietary Carbohydrates: New Insights From a Long Term Nutritional Study. *Aquaculture*.

[16] Cao J., Mei J., Xie J. (2024). Combined Effects of Hypoxia and Ammonia-N Exposure on the Oxygen Consumption, Glucose Metabolism and Amino Acid Metabolism in Hybrid Grouper (*Epinephelus fuscoguttatus♀ × E. lanceolatus♂*). *Veterinary Research Communications*.

[17] Khan I., Lu Y. N., Li N. (2023). Effect of Ammonia Stress on AMPK Regulating-Carbohydrate and Lipid Metabolism in Chinese Striped-Neck Turtle. *Comparative Biochemistry and Physiology C-Pharmacology Toxicology and Endocrinology*.

[18] Ji R. L., Xu X., Turchini G. M., Mai K. S., Ai Q. H. (2021). Adiponectin’s Roles in Lipid and Glucose Metabolism Modulation in Fish: Mechanisms and Perspectives. *Reviews in Aquaculture*.

[19] Jin J. H., Amenyogbe E., Yang Y. (2024). Effects of Ammonia Nitrogen Stress on the Physiological, Biochemical, and Metabolic Levels of the Gill Tissue of Juvenile Four-Finger Threadfin (*Eleutheronema tetradactylum*). *Aquatic Toxicology*.

[20] Wu Y. W., Zhao M. M., Xia Y. T. (2023). Deterioration of Muscle Quality Caused by Ammonia Exposure in Rainbow Trout (*Oncorhynchus mykiss*). *Food Bioscience*.

[21] Zou J. H., Hu P., Wang M. Y. (2023). Liver Injury and Metabolic Dysregulation in Largemouth Bass (*Micropterus salmoides*) After Ammonia Exposure. *Metabolites*.

[22] Wang T., Gao L., Li W. H., Li Y., Shan H. W. (2022). Comparative Transcriptome Analysis Reveals a Strategy Involving Dietary Manipulation for Reducing the Mortality of *Litopenaeus vannamei* Exposed to Sublethal Ammonia Through the Energy Metabolism Pathway. *Aquaculture International*.

[23] Li X., Wang S., Zhang M., Yu Y., Li M. (2022). Glutamine Synthetase (GS) Deficiency Can Affect Ammonia Tolerance of Yellow Catfish *Pelteobagrus Fulvidraco*. *Fish and Shellfish Immunology*.

[24] Gyamfi S., Edziyie R. E., Obirikorang K. A., Adjei-Boateng D., Skov P. V. (2024). Nile Tilapia (*Oreochromis niloticus*) Show High Tolerance to Acute Ammonia Exposure but Lose Metabolic Scope During Prolonged Exposure at Low Concentration. *Aquatic Toxicology*.

[25] Wang S. D., Li X., Zhang M. Z. (2024). Li, miR-199-5p Mediates the Regulation of Autophagy by Targeting mTOR Signaling and Involvement in Ammonia Detoxification Under Ammonia Stress in Yellow Catfish (*Pelteobagrus Fulvidraco*). *Aquaculture*.

[26] Xu Z. K., Cao J., Qin X. M., Qiu W. Q., Mei J., Xie J. (2021). Toxic Effects on Bioaccumulation, Hematological Parameters, Oxidative Stress, Immune Responses and Tissue Structure in Fish Exposed to Ammonia Nitrogen: A Review. *Animals-Basel*.

[27] Liang Y. S., Wu Y., Li J. P. (2022). Effects of Ammonia Toxicity on the Histopathology, Detoxification, Oxidative Stress, and Immune Response of the Cuttlefish *Sepia pharaonis* and the Mitigation of γ-Aminobutyric Acid. *Ecotoxicology and Environmental Safety*.

[28] Nakada T., Westhoff C. M., Kato A., Hirose S. (2007). Ammonia Secretion From Fish Gill Depends on a Set of Rh Glycoproteins. *The FASEB Journal*.

[29] Zhang M., Wang S., Li X., Li M. (2025). Activation Autophagy Enhances Ammonia Detoxification by Boosting Urea and Glutamine Synthesis in Yellow Catfish (*Pelteobagrus Fulvidraco*). *Aquaculture*.

[30] Soria L. R., Allegri G., Melck D. (2018). Enhancement of Hepatic Autophagy Increases Ureagenesis and Protects Against Hyperammonemia. *Proceedings of the National Academy of Sciences*.

[31] Jahanbani A., Shahriari A., Mohammadian T. (2025). The First Report of AMP-Deaminase Activity in Skeletal Muscles of *Lates calcarifer* and Its Stunning Adaptation to Ammonia Poisoning. *Fish Physiology and Biochemistry*.

[32] Chew S. F., Ip Y. K. (2014). Excretory Nitrogen Metabolism and Defence Against Ammonia Toxicity in Air-Breathing Fishes. *Journal of Fish Biology*.

[33] Ma Q., Luo Y., Zhong J. (2023). Hypoxia Tolerance in Fish Depends on Catabolic Preference Between Lipids and Carbohydrates. *Zool Res*.

[34] Su J. Z., Mei L. Y., Xi L. W. (2021). Responses of Glycolysis, Glycogen Accumulation and Glucose-Induced Lipogenesis in Grass Carp and Chinese Longsnout Catfish Fed High-Carbohydrate Diet. *Aquaculture*.

[35] Wang Z., Dong Z. D., Yang Y. T. (2022). Histology, Physiology, and Glucose and Lipid Metabolism of Lateolabrax Maculatus Under Low Temperature Stress. *Journal of Thermal Biology*.

[36] Jahanbani A., Shahriari A., Mohammadian T. (2023). Ureagenesis of Asian Seabass (*Lates calcarifer*) Under Ammonia Stress and Overcrowding. *Aquaculture*.

[37] Uddin K. B., Li Y., Zhang M. (2024). Various Effects of Feeding Level on Ammonia Tolerance in *Carassius auratus* Under Different Ammonia Exposure Stress and the Underlying Mechanisms. *Ecotoxicology and Environmental Safety*.

[38] Li Y., Li Y. C., Liu X. T. (2022). Blockage of Citrate Export Prevents TCA Cycle Fragmentation via Irg1 Inactivation. *Cell Reports*.

[39] Zhang Y. Q., Lai W. C., Chen Q. C. (2023). Calcium Pyruvate Attenuates Fat Deposition by Augmenting Fatty Acid Oxidation and Inhibiting Glucose Oxidation in Juvenile Large Yellow Croaker (*Larimichthys crocea*) Consuming a High-Fat Diet. *Aquaculture*.

[40] Nan Y. X., Xiao M., Duan Y. F., Yang Y. K. (2024). Toxicity of Ammonia Stress on the Physiological Homeostasis in the Gills of *Litopenaeus vannamei* Under Seawater and Low-Salinity Conditions. *Biology-Basel*.

[41] Sun H. R., Yue T. T., Hou Y. Q. (2025). Dietary Geniposide Supplementation Could Enhance Hepatic Lipid Metabolism, Immunity, Antioxidant Capacity, and Ammonia Stress Resistance in Turbot. *Animal Nutrition*.

[42] Zhang C., Huang W. H., Zhang X. T. (2024). Single and Combined Effects of High-Fat Diet and Ammonia Nitrogen on Oxidative Stress, Lipid Accumulation and Inflammation in Liver of Grass Carp. *Aquaculture*.

[43] Zhang C. Q., Chen W., Wang B. (2024). Potato Glycoside Alkaloids Exhibit Antifungal Activity by Regulating the Tricarboxylic Acid Cycle Pathway of Fusarium Solani. *Frontiers in Microbiology*.

[44] Sinha A. K., Liew H. J., Diricx M., Blust R., De Boeck G. (2012). The Interactive Effects of Ammonia Exposure, Nutritional Status and Exercise on Metabolic and Physiological Responses in Gold Fish (*Carassius auratus L*.). *Aquatic Toxicology*.

